# Active Iron Sites of Disordered Mesoporous Silica Catalyst FeKIL-2 in the Oxidation of Volatile Organic Compounds (VOC)

**DOI:** 10.3390/ma7064243

**Published:** 2014-05-30

**Authors:** Mojca Rangus, Matjaž Mazaj, Goran Dražić, Margarita Popova, Nataša Novak Tušar

**Affiliations:** 1 National Institute of Chemistry, Hajdrihova 19, SI-1001 Ljubljana, Slovenia; E-Mails: mojca.rangus@ki.si (M.R.); matjaz.mazaj@ki.si (M.M.); goran.drazic@ki.si (G.D.); 2 Institute of Organic Chemistry with Centre of Phytochemistry, Bulgarian Academy of Sciences, Acad. G. Bontchev str., bl. 9, 1113 Sofia, Bulgaria; E-Mail: mpopova@orgchm.bas.bg

**Keywords:** FeKIL-2, disordered mesoporous silica, interparticle mesoporosity, isolated iron, oligonuclear iron clusters, catalytic oxidation, VOC decomposition, XANES, EXAFS, AR-STEM

## Abstract

Iron-functionalized disordered mesoporous silica (FeKIL-2) is a promising, environmentally friendly, cost-effective and highly efficient catalyst for the elimination of volatile organic compounds (VOCs) from polluted air via catalytic oxidation. In this study, we investigated the type of catalytically active iron sites for different iron concentrations in FeKIL-2 catalysts using advanced characterization of the local environment of iron atoms by a combination of X-ray Absorption Spectroscopy Techniques (XANES, EXAFS) and Atomic-Resolution Scanning Transmission Electron Microscopy (AR STEM). We found that the molar ratio Fe/Si ≤ 0.01 leads to the formation of stable, mostly isolated Fe^3+^ sites in the silica matrix, while higher iron content Fe/Si > 0.01 leads to the formation of oligonuclear iron clusters. STEM imaging and EELS techniques confirmed the existence of these clusters. Their size ranges from one to a few nanometers, and they are unevenly distributed throughout the material. The size of the clusters was also found to be similar, regardless of the nominal concentration of iron (Fe/Si = 0.02 and Fe/Si = 0.05). From the results obtained from sample characterization and model catalytic tests, we established that the enhanced activity of FeKIL-2 with the optimal Fe/Si = 0.01 ratio can be attributed to: (1) the optimal concentration of stable isolated Fe^3+^ in the silica support; and (2) accelerated diffusion of the reactants in disordered mesoporous silica (FeKIL-2) when compared to ordered mesoporous silica materials (FeSBA-15, FeMCM-41).

## 1. Introduction

Volatile organic compounds (VOCs) comprise the main class of air pollutants emitted from various industrial processes [1]. Catalytic oxidation is one of the most important industrially applicable processes for the decomposition of VOCs in polluted air. The advanced VOC removal process is composed of an adsorption unit, followed by a catalytic incinerator. One of the alternatives to noble metal-containing catalysts currently used for this process is transition metal oxides or other species immobilized on a suitable support. The nature of the support and the applied method of immobilization are important factors in the preparation of a catalyst, as its surface area and the nature and dispersion of the metal species determine the material’s catalytic behavior and functionality. During the last decades, ordered mesoporous silica supports (MCM-41, SBA-15, SBA-16) have been of particular interest, because of their uniform mesoporous channel structure and their high specific surface area [2,3]. We have recently shown that disordered mesoporous silica (KIL-2) with a high specific surface area can be used successfully as a catalytic support for immobilizing iron (FeKIL-2) [4]. KIL is a group of materials first synthesized at the National Institute of Chemistry in Ljubljana (Kemijski Inštitut Ljubljana—KIL) [5]. It is composed of agglomerated silica nanoparticles and has interparticle porosity. FeKIL-2 is low cost and has also proven to be a highly efficient catalyst for the removal of VOCs; it also shows great potential for air purification applications [4]. However, a more detailed investigation of catalytically active species and their presence depending on the concentration or Fe in the matrix has not yet been done. The results of the basic FeKIL-2 characterization (UV/Vis, FTIR and Mössbauer spectroscopic techniques) combined with its catalytic performance suggest that the activity of the FeKIL-2 catalyst can be attributed to the formation of stable isolated Fe^3+^ ions in the silica matrix, ensuring easier oxygen release from the catalyst (Fe^3+^/Fe^2+^ redox cycles) [4]. In the same paper, it was also determined that the sample with Fe/Si = 0.01 molar ratio showed the best catalytic performance.

The local environment of metal atoms incorporated in porous silicates is typically investigated by different spectroscopic techniques. The choice of the spectroscopic method is governed by the nature of the incorporated metal. Besides UV-VIS spectroscopy, IR (Infrared) spectroscopy, Mössbauer spectroscopy and X-ray photoelectron spectroscopy (XPS), the spectroscopic techniques most commonly used for this type of study, as well as being X-ray absorption spectroscopy methods, are namely Extended X-ray Absorption Fine Structure (EXAFS) and X-ray Absorption Near Edge Structure (XANES). The combined use of these techniques with, for example, atomic-resolution scanning transmission electron microscopy (AR STEM) can lead to a reliable characterization of iron sites.

There has been some discussion in the literature on the presence of different iron species in crystalline microporous silica (FeZSM-5: isolated iron, oligonuclear iron clusters and iron-oxides) [6,7,8,9], amorphous ordered mesoporous silica (FeSBA-15, FeMCM-41: iron oxides) [10,11,12] and amorphous disordered mesoporous silica (FeTUD-1: isolated iron) [13]. Different iron species are also associated with different catalytic reactions [6,7,8,9,10,11,12,13].

In this study, we focused on (1) studying the form and size of iron species formed in disordered mesoporous silica, FeKIL-2, depending on the iron concentration and (2) determining why the sample with Fe/Si = 0.01 molar ratio shows the best catalytic performance. The spectroscopic investigation was done using XANES, EXAFS and AR-STEM (Z-contrast imaging and Electron Energy Loss Spectroscopy or EELS) techniques. We also discuss the catalytic activity of FeKIL-2 with different Fe/Si molar ratios and compare the best performing FeKIL-2 sample with Fe/Si = 0.01 to the iron functionalized ordered mesoporous silica matrices (FeMCM-41, FeSBA-15) in the catalytic oxidation of toluene as a model VOC.

## 2. Results and Discussion

It has already been shown that metal-modified disordered mesoporous KIL-2 performs well in advanced oxidation processes (AOP) for water [14] and air purification from organic pollutants [4]. For the current study, catalysts for VOC removal from air were prepared by incorporating iron into the KIL-2 matrix. To elucidate why 0.01 is the optimal Fe concentration for this reaction, samples with various Fe/Si molar ratios of 0.005, 0.01, 0.02 and 0.05 were prepared. We were particularly interested in identifying catalytically active sites in the FeKIL-2 samples and explaining the observed variation in the catalytic activity for different iron loadings. The textural properties of pure and Fe-modified KIL-2 samples obtained from nitrogen sorption isotherm data are given in Table 1. The results of the BET surface area and pore volume values seem to be inconsistent with the amount of incorporated iron within the KIL-2 matrix. The highest specific surface area and the largest pores were calculated for the sample with an Fe/Si molar ratio of 0.01.

**Table 1:** BET surface areas (*S*_BET_) and total pore volumes (*V*_t_) evaluated from N_2_ adsorption isotherms at 0.98 relative pressure [4].

Sample	*S*_BET_ (m^2^/g)	*V*_t_ (cm^3^/g)
KIL-2	545	1.480
005FeKIL-2	472	1.292
01FeKIL-2	556	1.459
02FeKIL-2	213	0.976
05FeKIL-2	366	0.640

**Figure 1:**
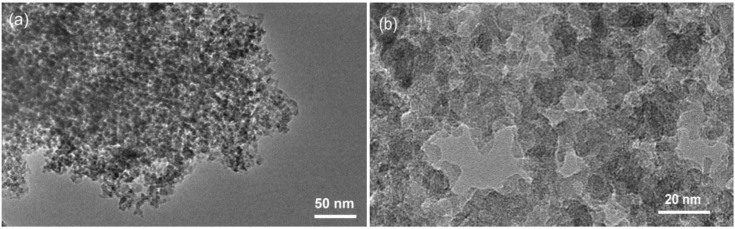
(**a**) Transmission electron microscopy (TEM) micrographs of 02-FeKIL-2 agglomerate; (**b**) close-up image of 02-FeKIL-2 material showing a typical KIL-2 nanostructure.

With transmission electron microscopy (TEM) a typical KIL-2 silica matrix with disordered mesoporous structure was observed (Figure 1). Amorphous silica nanoparticles form mesopore openings with a broad range of dimensions from 5 nm up to approximately 50 nm. Observations at higher magnification did not reveal the presence of any extra-framework iron oxide nanoparticles, indicating that iron is most probably incorporated within the silica framework either as isolated Fe cations or as bigger Fe oligomeric chains.

**Figure 2:**
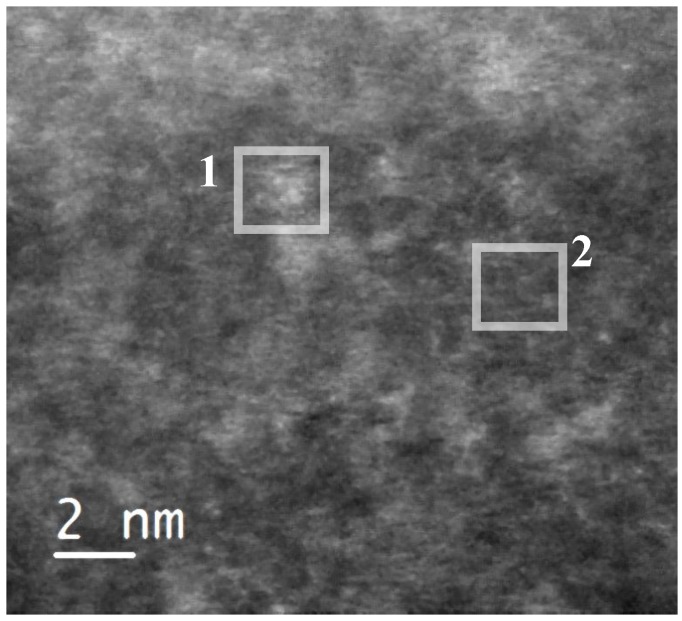
High-angle annular dark field (HAADF) micrograph of a 05FeKIL-2 silica particle, where one to two nm sized areas with brighter contrast could be seen. Squares indicate the positions where EELS spectra were collected.

**Figure 3:**
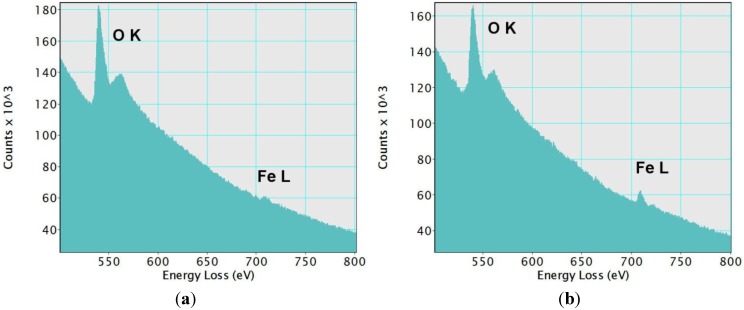
Electron Energy Loss Spectroscopy (EELS) spectra from (**a**) region 1 and (**b**) region 2 in Figure 2. A small L3 peak at the edge threshold can be noticed in the spectrum from region 1. In region 2, only traces of iron were detected. In both cases, the O_K_ peaks were clearly observed.

In order to identify the observed iron species, the sample was scanned in high-angle annular dark field scanning transmission electron mode (HAADF-STEM) (Figure 2). This imaging technique shows some bright domains on the silica matrix, which has a dark grey color. We estimated the average size of these domains to be 1.5 nm. They indicate the presence of elements with an atomic number higher than silicon (Z-contrast). They could correspond to the iron-based species incorporated in the silica framework. Both the lighter and darker areas of the sample were analyzed by EELS spectroscopy Figure 3. As expected, peaks characteristic of oxygen and iron were found in the brighter areas (Figure 3a). On the other hand, darker Z-contrast regions showed only traces of iron (Figure 3b). In the EELS spectrum of iron, two sharp peaks L2, 3 at the edge threshold around 709 eV are normally observed. Due to the very small concentration of iron in the examined samples, the L3 peak was barely observable even in the lighter-colored areas (Figure 3a). The results obtained with the STEM/EELS analysis clearly indicate that iron in the sample is incorporated into the silica framework in the form of iron-rich domains or islands. Such clusters were observed in samples with Fe/Si molar ratios of 0.02 and 0.05. By taking into account the dimensions of these observed domains, oligomers can be defined as two- or three-dimensional (FeO_4_)*_n_* (*n* = 3–6) species. Interestingly, the size of these iron-rich islands remains the same regardless of the Fe content (0.02 or 0.05).

For a further and more detailed study of iron species in the FeKIL-2 samples, the use of X-ray absorption spectroscopy (XAS) was employed. The aim was to try to understand how and why the amorphous KIL-2 structure along with the iron concentration influence the incorporation of iron ions into the matrix. X-ray absorption spectroscopy probes the local environment of observed atoms, determining their coordination, type and the distance of the nearest neighboring atoms and degree of order or disorder. It is also possible to determine the average oxidation state of observed ions in the samples. In the XANES part of the absorption spectra, the shape of the K-edge and the pre-edge features are characteristic of the local symmetry of the investigated atom. With increasing oxidation, the energy position of the absorption edge and the pre-edge resonances shift towards higher energies [15,16,17]. For the K adsorption edge, a linear relationship between the energy position and the average valence state was established for atoms with the same type of ligands [15,16]. Moreover, the energy positions of iron pre-edge features also strongly depend on the Fe oxidation state [18,19]. In addition to the absorption edge position, this characteristic can be used to determine the valence state of iron in the sample. For iron atoms coordinated to oxygen atoms, the reported energy shifts are approximately 4.5 eV per oxidation state for the iron K-edge [20,21] and 1.5 eV per oxidation state for the pre-edge feature [18,19]. Since not only the position, but also the shape of the absorption edge changes with different local geometries and ligand types, one must take great care when determining the oxidation state of observed ion species. Reference compounds with the same ligand types and coordination to the samples must be chosen to make a valid evaluation of the valence states.

As well as determining the iron oxidation state from the energy shift, the shape and intensity of the pre-edge features are characteristic of the coordination of the iron ions. Wilke *et al.* [18,19] also showed that the integrated pre-edge peak intensity is a good indicator of iron atom coordination. Iron ions in a tetrahedral environment have the highest pre-edge resonance intensity. A single pre-edge resonance with a 10% intensity of the edge jump is characteristic of this coordination. A distorted tetrahedral coordination also decreases the intensity of the pre-edge resonance. A lower intensity single pre-edge peak is attributed to 5-fold coordinated Fe atoms, while octahedrally coordinated iron atoms exhibit two weak resonances in the pre-edge region [18,19,20,22,23]. In addition, a mixture of 4- and 5-fold or 4- and 6-fold coordinated iron atoms also lowers the intensity of the pre-edge resonance.

**Figure 4:**
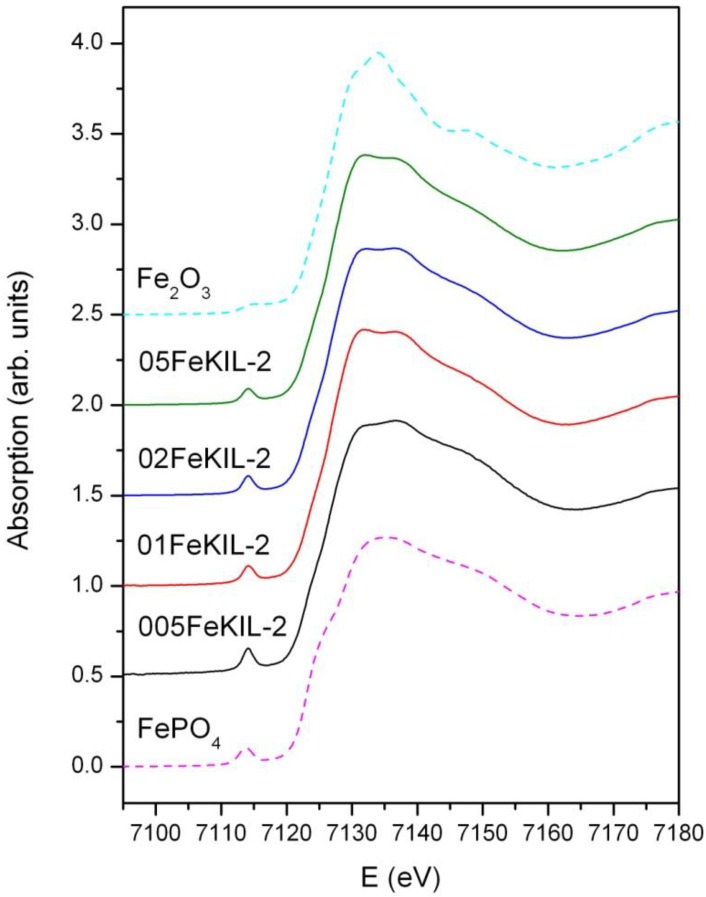
Normalized Fe K-edge X-Ray absorption near edge structure (XANES) data of FeKIL-2 samples and Fe reference compounds: Fe_2_O_3_ (octahedrally coordinated iron 3+ ions) and FePO_4_ (ferric ions in a tetrahedral coordination).

**Figure 5:**
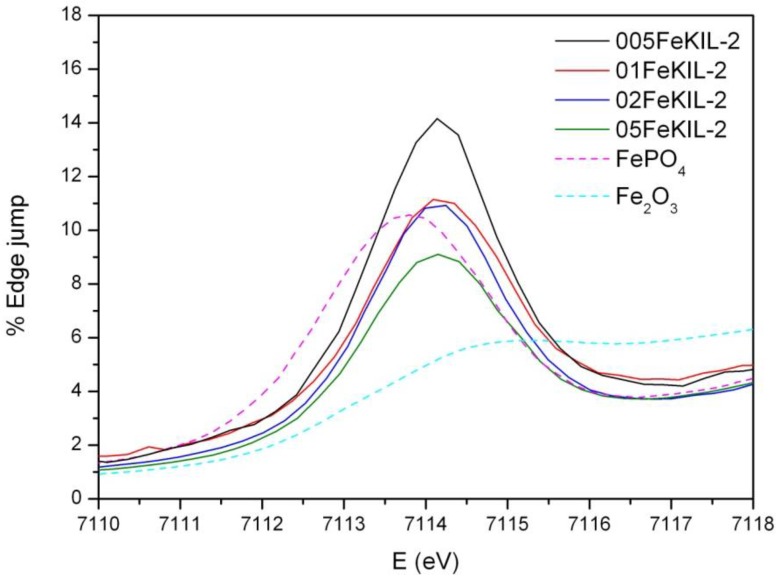
Fe K-edge X-ray absorption spectra of the FeKIL samples and reference compounds of the pre-edge region. A comparison of the heights and shapes of the pre-edge resonances.

We determined the average oxidation state of iron ions in our samples by comparing their Fe K-edge positions (Figure 4) and the energy positions of the pre-edge features (Figure 5) to the edge positions of the reference compounds with known valence states. We were able to assign the valence number of iron in the sample to be 3+, although we cannot completely exclude the possibility of the presence of some Fe^2+^ ions.

To determine the Fe ion coordination, we compared the pre-edge XANES spectra of the FeKIL-2 samples to a FePO_4_ model compound which has a symmetric tetrahedral coordination of iron ions (Table 2). All FeKIL-2 samples exhibit a strong single pre-edge peak with intensity comparable to FePO_4_, indicating a tetrahedral symmetry. The tetrahedral coordination of iron ions in micro- and mesoporous silicates is typical of framework iron atoms [13,24], *i.e.*, isolated Fe^3+^ sites and oligomeric iron species. In crystalline silica matrices [24] the incorporation of iron in higher concentrations results in the formation of oxides. However, in the case of a disordered FeKIL-2 structure, iron is incorporated in the matrix even at high concentrations such as 0.02 and 0.05. Similar results were obtained for the structurally similar (disordered mesoporous matrix with interparticle porosity) FeTUD-1 [13]. In the case of 05FeKIL-2 (the sample with the highest iron concentration) the lower intensity of the pre-edge resonance might indicate the presence of a small fraction of 5-fold coordinated Fe ions. However, XANES spectra of all the studied samples suggest that a higher than four-fold iron coordination seems not to be favored.

**Table 2:** Fe K-edge X-ray absorption near edge structure (XANES) data on the pre-edge resonance position and intensity for the FeKIL-2 samples.

Sample	Intensity (%)	Pre-peak position (eV)
005FeKIL-2	14.2	7114.1
01FeKIL-2	11.2	7114.1
02FeKIL-2	10.9	7114.1
05FeKIL-2	9.1	7114.1
FePO_4_	10.6	7113.8

**Figure 6:**
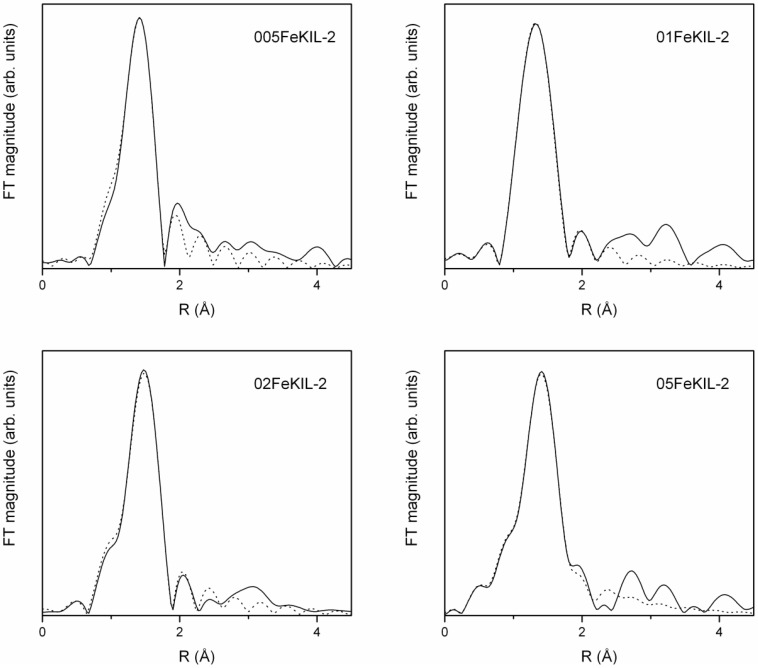
Fourier transform magnitudes of the *k*^3^-weighed Fe extended X-Ray absorption fine structure (EXAFS) data of FeKIL-2 samples (solid line—experiment; dotted line—EXAFS model).

The Fe K-edge EXAFS part of the FeKIL-2 X-ray absorption spectra was analyzed to produce an even more detailed image of the immediate Fe ion surroundings. The quantitative analysis of the *k*^3^-weighted Fe EXAFS data was performed with the IFEFFIT program packages [25] using FEFF6 code [26]. The k range of 3.2–12.0 Å^−1^ for 005FeKIL-2, 3.5–11.0 Å^−1^ for 01FeKIL-2, 3.5–13.5 Å^−1^ for 02FeKIL-2 and 3.2–11.4 Å^−1^ for 05FeKIL-2 samples was used. Only the first iron coordination sphere in the range of 1–2.4 Å was fitted. Fe K-edge EXAFS data of the FeKIL-2 samples along with the best fit EXAFS models (Figure 6) give a description of the short-range order of the iron atoms in terms of neighbors, distances and thermal and static disorder. A complete list of these best fit parameters is given in Table 3.

**Table 3:** Structural parameters of the nearest coordination shells around Fe atom in the FeKIL-2 samples: type of neighbor atom, average number N, distance R, and Debye-Waller factor σ^2^. Uncertainties in the last digit are given in parentheses.

Samples	Neighbor	N	R (Å)	σ^2^ (Å^2^)
**005FeKIL-2**	O	3.4(7)	1.83(2)	0.003(3)
	O	0.6(7)	2.0(1)	0.003(3)
**01FeKIL-2**	O	3	1.86(1)	0.003(1)
	O	1	2.04(4)	0.003(1)
**02FeKIL-2**	O	3.8(9)	1.87(2)	0.003(2)
	O	0.2(9)	2.0(4)	0.003(2)
**05FeKIL-2**	O	2.8(6)	1.88(3)	0.005(3)
	O	1.2(6)	2.04(4)	0.005(3)

EXAFS analysis of the FeKIL-2 samples shows a distorted tetrahedral environment of Fe ions consistent with the literature [8,13]. A first-shell model with oxygen atoms at two different distances had to be used to successfully fit the spectra. For all FeKIL-2 samples, Fe cations were found to be tetrahedrally coordinated with four oxygen atoms, three framework at a shorter distance of 1.83–1.88 Å and one O atom at a greater distance of approximately 2.0 Å. Such coordination is characteristic of isolated iron sites or oligonuclear iron clusters, and suggests their incorporation into the silicate framework [8,13,24]. Bordiga *et al.* [8] suggest this is due to the –Si–OH–Fe– species with Fe–O(H) distance larger than the other three Fe–O. Since the KIL-2 structure is completely disordered, the second coordination shell of these iron ions is not well determined and ordered. However, a degree of disorder in the Fe environment beyond 2.0 Å cannot be attributed to the nature of the matrix alone, and points to the presence of numerous different Fe^3+^ species and oligomers. At the same time, it excludes the presence of iron oxides, since even nanoparticles several nm in diameter would result in strong signals at distances beyond 2 Å. Taking this into account, we focused the analysis on the first iron coordination shell in order to note any changes in Fe–O distances and the oxygen Debye-Waller factors for samples with different Fe loadings. We would also like to emphasize that EXAFS analysis gives only an average picture of, in this case, the highly disordered iron environment in the samples. Therefore, we were unable to estimate or distinguish the contributions of different framework Fe species. We would also like to point out that, despite the calcination of the samples prior to the analyses, Fe remained 4-fold coordinated. This confirms the structural stability of the samples even when exposed to temperatures as high as 500 °C for a number of hours. As a comparison; in the case of disordered mesoporous silicate FeTUD-1, iron atoms that were tetrahedrally coordinated in the as-synthesized sample became octahedrally coordinated after calcination [13].

**Figure 7:**
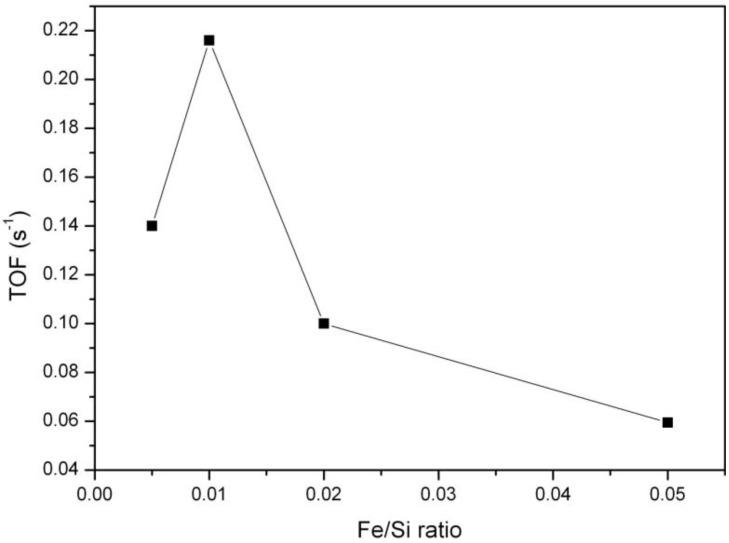
Dependence of catalytic activity (on stream toluene oxidation after 60 min at a constant temperature of 380 °C) upon the iron concentration for the FeKIL-2 samples.

Catalytic oxidation of toluene was chosen as a model reaction to test FeKIL-2 materials for their catalytic performance in the decomposition of volatile organic compounds [4]. The simultaneous presence of Fe^2+^/Fe^3+^ species in the catalyst during the reaction provides easier oxygen release. This is essential for the Mars-van Krevelen mechanism [27,28], widely accepted in the literature for this type of reaction. The mechanism of oxidation suggests the adsorption of the VOC molecule on the catalyst surface, its oxidation with lattice oxygen following the oxidation of the reduced catalysts. The process of adsorption of organic molecules can be affected by the surface properties of the catalysts. Activity in total oxidation of VOC is connected with the interaction of aromatic electrons with metal ions in the tetrahedral position acting as Lewis and/or Brönsted acid sites, increasing the possibility of an electrophilic attack of adsorbed oxygen and combustion of toluene molecules. Toluene oxidation at a constant temperature of 380 °C and after 60 min on stream as a function of iron concentration in the samples is presented in Figure 7. It shows that catalytic activity first increases with increasing iron concentration in the samples, but for Fe/Si ratios higher than 0.01 it starts to decrease. Evidently, there is a maximum of catalytic activity around Fe/Si = 0.01. A much more detailed catalytic study including a larger variety of Fe/Si ratios would be needed to identify a material with the optimal Fe loading with a higher degree of certainty.

**Figure 8:**
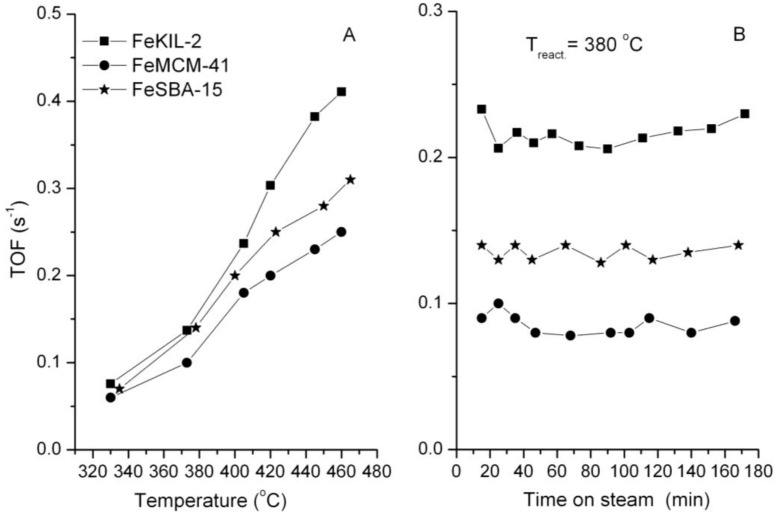
.Catalytic activity *vs.* (**A**) temperature; and (**B**) time on stream at 380 °C on FeKIL-2, FeMCM-41, FeSBA-15 samples (Fe/Si = 0.01). Operating conditions: *T* = 300–480 °C (**A**); atmospheric pressure, *p*_toluene_ = 0.9 kPa; WHSV = 1.2 h^−1^.

The best catalytically performing FeKIL-2 sample, 01FeKIL-2, shows higher catalytic activity compared to ordered mesoporous silica FeMCM-41 and FeSBA-15 with the same Fe/Si molar ratio (Figure 8). We attribute this to the predominate incorporation of isolated Fe^3+^ ions into the silica matrix for FeKIL-2 [4], which favors the fast Fe^3+^ ↔ Fe^2+^ transition essential for the oxidation reaction. In addition, because of its disordered structure FeKIL-2 also possesses a better diffusion characteristic compared to ordered mesoporous silicates. In the case of iron containing FeMCM-41, the lower catalytic activity could also be assigned to the partial iron release from the tetrahedral position, most probably with the formation of hematite-like nanoparticles [2]. A constant catalytic performance in the course of three hours (Figure 8B) shows that all the samples remained unaltered and their characteristics unchanged.

In the case of FeKIL-2, we can see how the iron concentration along with a disordered silica structure influences the catalytic activity. The increase in catalytic activity up to a certain Fe/Si molar ratio, followed by a decrease in catalytic activity for a higher Fe concentration in the KIL-2 silica matrix (Figure 7), could be simply related to the textural properties of the modified materials. The best catalytic performance material was shown to be 01FeKIL-2, which also has the highest surface area and mesopore volume values of all the investigated samples. This enables the most effective diffusivity for host molecules. On the other hand, the catalytic activity of the sample per S_BET_ or V_t_ follows roughly the same trend as presented in Figure 7, with the optimal Fe/Si ratio around 0.01. Therefore, the optimal catalytic performance at a certain amount of Fe within the silica matrix can also be explained by the following hypothesis. HAADF-STEM analysis showed that the majority of iron atoms are accumulated in the Fe-rich islands. Since KIL-2 structure is completely disordered, iron ions during the synthesis process are randomly distributed in these domains. So, the higher the iron concentration in the sample, the shorter the special distances between the Fe atoms are. As was already reported for disordered silica with interparticle porosity, the as-synthesized samples contain only four-fold coordinated Fe^3+^ ions in a symmetric tetrahedral environment [13]. As the sample is calcined, isolated iron sites that are close enough migrate out of position and towards each other and form oligomeric iron clusters [24,29]. The presence of two types of framework iron with different catalytic activity in the microporous silicates was demonstrated by Kumar *et al.* [24]. However, if the iron atoms are too far apart, they remain in the framework as isolated Fe^3+^ sites. We believe that for this reason there are only isolated iron sites present in the samples with low iron concentration (*i.e.*, 005FeKIL-2). Since the 01FeKIL-2 sample contains an Fe/Si ratio two times higher than the 005FeKIL-2 sample, it has about twice the amount of catalytically active Fe^3+^ sites. Some iron oligomeric clusters may also form during calcination, but we believe that at Fe/Si = 0.01 iron content, the vast majority of the iron occupies the isolated sites. Hence the catalytic activity of the 01FeKIL-2 sample increases. In the case of 02FeKIL-2 and 05FeKIL-2 samples, the spatial distance of randomly distributed iron atoms is short enough for more iron oligomers to be formed. Since (FeO_4_)*_n_* oligomers are not catalytically active species in the studied reaction [4] and only some isolated iron sites still remain, the catalytic activity for these samples decreases. As iron atoms seem to be concentrated in iron-rich areas, they are much closer together, and oligomeric clusters form at much lower Fe content than if the Fe atoms were evenly distributed throughout the entire sample. On the other hand, since KIL-2 has a completely disordered nanostructure and thus has more defect sites, it can more easily accommodate larger amounts of isolated Fe ions than ordered mesoporous silicates (SBA-15 or MCM-41). A higher Fe accommodation capacity could also be the reason we could not detect any iron oxides in the FeKIL-2 samples. Along with the better diffusion characteristics, this could be the reason for the better toluene oxidation performance of FeKIL-2.

## 3. Experimental Section

### 3.1. Synthesis Procedure

We incorporated iron with Fe/Si molar ratios from 0.005 to 0.05 via a two-step solvothermal synthesis into a silicate matrix from the KIL family (KIL-2) [4]. KIL-2 is formed from amorphous silica nanoparticles (10–20 nm) with interparticle mesoporosity [5]. We denoted the prepared catalysts as 005FeKIL-2 (Fe/Si = 0.005), 01FeKIL-2 (Fe/Si = 0.01), 02FeKIL-2 (Fe/Si = 0.02) and 05FeKIL-2 (Fe/Si = 0.05). Removal of the template was performed by calcination in air at 500 °C.

### 3.2. Catalytic Test

Prior to the catalytic test, the samples were pretreated for 1 h in air up to 450 °C. Toluene oxidation was studied at atmospheric pressure using a fixed-bed flow reactor with air (30 cm^3^/min) as carrier gas in the temperature interval of 350–450 °C at WHSV of 1.2 h^−1^. In the reaction 30 mg sample (particle size 0.2–0.8 mm) was tested, diluted with 60 mg glass beads of the same diameter, previously checked to be inactive. The reactor itself was a quartz tube of 15 mm inner diameter, with the catalyst bed at the middle. A thermocouple was positioned in the catalyst bed for accurate measurement of the catalyst temperature. All gas lines of the apparatus were heated continuously at 383 K in order to minimize VOC adsorption on the tube walls. The air stream passed through a saturator filled with toluene and equilibrated at 0 °C (*p*_toluene_ = 0.9 kPa). The reaction steady state was established after 30 min in each temperature. On-line analysis of the reaction products was performed using HP-GC with a 25 m PLOT Q capillary column. The turnover frequency (TOF) was calculated as the converted number of toluene molecules per metal atom per second. The catalysts keep their catalytic activity over three reaction cycles.

### 3.3. Characterization of Local Environment of Iron

The X-ray absorption spectra of analyzed materials and reference compounds were measured in the Fe K-edge energy region (7112 eV) in the transmission detection mode at beamline C at the HASYLAB synchrotron facility at DESY in Hamburg, Germany and at the XAFS beamline of the ELETTRA synchrotron facility in Basovizza, Italy. At both beamlines, a Si(111) double crystal monochromator was used with approx. 1 eV energy resolution at the Fe K-edge. Higher harmonics were effectively eliminated by a slight detuning of the second monochromator crystal, keeping the intensity at 60% of the rocking curve with the beam stabilization feedback control. The intensity of the monochromatic X-ray beam was measured by three consecutive ionization chambers filled with (1) 950 mbar N_2_; (2) 350 mbar Ar; (3) 530 mbar Ar at HASYLAB and (1) 580 mbar N_2_ and 1420 mbar He; (2) 90 mbar Ar, 1000 mbar N_2_ and 910 mbar He; (3) 350 mbar Ar, 1000 mbar N_2_ and 650 mbar He at ELETTRA. The samples and the reference compounds were prepared as homogeneous self-supporting pellets, with a total absorption thickness (μd) of approx. 2.5 above the Fe K-edge and mounted on a sample holder between the first and second ionization detectors. The absorption spectra of the samples were measured in the energy region from 250 eV before to 1000 eV above the Fe K-edge, with an integration time of 1 s/step. In the XANES region, equidistant energy steps of 0.25 eV were used, while for the EXAFS region, equidistant *k*-steps (*k* = 0.03 Å^−1^) were adopted. Exact energy calibration was established with simultaneous absorption measurements on Fe metal foil inserted between the second and third ionization cells.

Transmission electron microscopy (TEM) was performed on a 200 kV cold field-emission gun (FEG) Cs-probe corrected transmission electron microscope (Jeol ARM 200 CF), coupled with a Gatan Quantum ER electron energy-loss spectroscopy (EELS) system and energy-dispersive X-ray spectrometry (Jeol Centurio 100). Samples were dispersed in ethanol and placed on a holey carbon coated copper grid. The specimens were additionally coated with carbon to prevent excessive charging of the samples under the electron beam.

## 4. Conclusions

Catalytic tests indicate that disordered mesoporous structures show potential in catalytic oxidation of VOCs. They have a higher capacity for the incorporation of iron in the silica matrix and have better diffusion characteristics compared to the ordered mesoporous silicates. Analyses of the FeKIL-2 samples by a combination of X-ray Absorption Spectroscopy Techniques and Atomic Resolution Electron Microscopy firstly dismissed the existence of iron oxide nanoparticles and secondly confirmed the presence of iron oligomers and isolated Fe sites. The tetrahedrally coordinated isolated Fe^3+^ species participates in the toluene oxidation process [4] and its presence is important for a successfully performing catalyst. It was found that the Fe/Si molar ratio in the proximity of 0.01 leads to the formation of stable isolated Fe^3+^ sites. Higher iron content (Fe/Si >0.01) leads to the formation of oligonuclear iron clusters from one to a few nanometers in size, which were unevenly distributed in the material, changing the morphology of the samples and hindering their catalytic performance.

The nature of iron species, along with the disordered mesoporous silica matrix of FeKIL-2, yielded a promising, environmentally friendly, cost-effective and highly efficient catalyst, for the elimination of VOCs from polluted air via catalytic oxidation.
